# Risk of Dementia or Cognitive Impairment in Sepsis Survivals: A Systematic Review and Meta-Analysis

**DOI:** 10.3389/fnagi.2022.839472

**Published:** 2022-03-09

**Authors:** Siyuan Lei, Xuanlin Li, Hulei Zhao, Zhenzhen Feng, Liu Chun, Yang Xie, Jiansheng Li

**Affiliations:** ^1^Department of Respiratory Diseases, Longhua Hospital Shanghai University of Traditional Chinese Medicine, Shanghai, China; ^2^Co-construction Collaborative Innovation Center for Chinese Medicine and Respiratory Diseases by Henan and Education Ministry of P.R. China, Henan University of Chinese Medicine, Zhengzhou, China; ^3^Department of Respiratory Diseases, The First Affiliated Hospital of Henan University of Chinese Medicine, Zhengzhou, China

**Keywords:** sepsis, dementia, cognitive impairment, meta-analysis, systematic review

## Abstract

**Background:**

There is growing evidence that sepsis survivors are at increased risk of developing new-onset atrial fibrillation, acute kidney injury, and neurological diseases. However, whether sepsis survivals increase the risk of dementia or cognitive impairment remains to be further explored.

**Objective:**

The objective of this study was to determine whether sepsis survivals increase the risk of dementia or cognitive impairment.

**Methods:**

We searched PubMed, Cochrane Library, Web of Science, and EMBASE databases for cohort studies or case-control studies from their inception to November 5, 2021. The quality of this study was assessed using the Newcastle-Ottawa Quality Assessment Scale (NOS). The Stata software (version 15.1) was used to calculate the odds ratio (OR) of dementia or cognitive impairment in sepsis survivals. Subgroup and sensitivity analyses were performed to assess the source of heterogeneity. Funnel plots and Egger’s test were used to detect the publication bias.

**Results:**

Eight studies (i.e., seven cohort studies and one case-control study) involving 891,562 individuals were included. The quality assessment results showed that the average score of NOS was over 7, and the overall quality of the included studies was high. Pooled analyses indicated that sepsis survivals were associated with an increased risk of all-cause dementia (OR = 1.62, 95% CI = 1.23–2.15, *I*^2^ = 96.4%, *p* = 0.001). However, there was no obvious association between sepsis survivals and the risk of cognitive impairment (OR = 1.77, 95% CI = 0.59–5.32, *I*^2^ = 87.4%, *p* = 0.306). Subgroup analyses showed that severe sepsis was associated with an increased risk of dementia or cognitive impairment (OR = 1.99, 95% CI = 1.19–3.31, *I*^2^ = 75.3%, *p* = 0.008); such risk was higher than that of other unspecified types of sepsis (OR = 1.47, 95% CI = 1.04–2.09, *I*^2^ = 97.6%, *p* = 0.029).

**Conclusion:**

Sepsis survivals are associated with an increased risk of all-cause dementia but not with cognitive impairment. Appropriate management and prevention are essential to preserve the cognitive function of sepsis survivors and reduce the risk of dementia.

## Introduction

Sepsis is a life-threatening organ dysfunction resulting from a dysregulated host response to infection (Evans et al., 2021), which has become a leading cause of death and critical illness worldwide (Vincent et al., 2014; Fleischmann et al., 2016). It is characterized by high morbidity and mortality, and more than 30 million cases occur annually worldwide, including at least 6 million deaths (WHO, 2018). A recent meta-analysis showed an incidence of 189 cases of hospital-treated adult sepsis per 100,000 person years and a mortality rate of 26.7% (Fleischmann-Struzek et al., 2020). Sepsis remains a heavy burden across all economic regions and was reported to account for more than US$23.7 billion (6.2%) of total hospital costs in the United States in 2011, which may be higher in low-level income countries (Seymour et al., 2012; Torio and Andrews, 2013). The Global Sepsis Alliance (2015) stated that at least 20% of sepsis survivors experience sequelae, such as physical or cognitive impairment and mood disorders, which result in poor quality of life (Iwashyna et al., 2010; Global Sepsis Alliance 2015). The current research suggests that approximately 3 million patients with sepsis whose electroencephalogram (EEG) activity continues to slow down, memory is impaired, and hippocampal volume is reduced (Semmler et al., 2013) will survive with cognitive and functional impairments per year (Perner et al., 2018). Therefore, more attention should be paid to the cognitive function in sepsis survivals.

Dementia is regarded as a global public health problem, affecting around 50 million individuals and resulting in a cost of 1% of global gross domestic product (GDP) (Addressing global dementia, 2014). It is estimated that the number of individuals who develop dementia will rise to 131.5 million by 2050 owing to aging populations and the lack of effective preventive measures (Alzheimer’s Disease International, 2015). Researchers have explored the association of inflammatory markers with dementia or cognitive impairment, and bacterial infection has been shown as a potential risk factor for dementia (Maheshwari and Eslick, 2015; Sochocka et al., 2017). Notably, sepsis is a syndrome resulting from severe bacterial infections.

There is increasing evidence that sepsis survivals are at an increased risk of developing new-onset atrial fibrillation (Xiao et al., 2021), acute kidney injury (Liu et al., 2020), and neurological diseases (Axer et al., 2014). However, whether sepsis survivals increase the risk of dementia or cognitive impairment remains unclear. Previous studies (Shen et al., 2012; Kao et al., 2015; Liao et al., 2015; Bouza et al., 2019) have found that dementia increases the risk of acute organ dysfunction and sepsis in hospitalized elderly patients. In addition, several observational studies (Iwashyna et al., 2010; Guerra et al., 2012; Shah et al., 2013; Mawanda et al., 2016; Chou et al., 2017; Sakusic et al., 2018; Ahlström et al., 2020; Fritze et al., 2020) have recently explored the risk of dementia or cognitive impairment in sepsis survivals. However, their conclusions were inconsistent, and their exact nature remains uncertain. Therefore, we conducted a systematic review and meta-analysis on the existing population-based longitudinal evidence to determine whether sepsis survivals are associated with an increased risk of dementia or cognitive impairment.

## Methods

This study was conducted in accordance with the Preferred Reporting Items for Systematic Reviews and Meta-Analyses (PRISMA) guidelines (Page et al., 2021), and the protocol has been registered in the International Prospective Register of Systematic Reviews (PROSPERO) (CRD42021292172) (Lei et al., 2021).

### Search Strategy

We systematically searched the PubMed, EMBASE, Cochrane Library, and Web of Science databases without language restrictions from their inception to November 15, 2021. The Medical Subject Headings (MeSH) terms and keywords used in the search were as follows: (“sepsis” OR “septicemia*” OR “septic shock*” OR “severe sepsis*” OR “systemic inflammatory response syndrome” OR “SIRS” OR “septic” OR “septicaemic shock”) AND (“dementia” OR “Alzheimer’s disease” OR “cognitive decline” OR “cognitive impairment” OR “cognitive disorder” OR “cognitive dysfunction”). The references of the included studies and existing systematic reviews were hand-searched to find additional relevant articles. The full search strategy is included in Supplementary Appendix 1.

### Eligibility Criteria

The included studies were required to meet the following criteria: (1) cohort or case-control study design; (2) the exposed group consisting of sepsis survivals with dementia or cognitive impairment, and the control group consisting of patients without sepsis; (3) the risk of dementia or cognitive impairment as the outcome, expressed as an adjusted odds ratio (OR); and (4) population-based study design. In this study, all types of dementia, such as vascular dementia, senile dementia, Alzheimer’s disease, and mixed dementia, were included.

### Exclusion Criteria

Exclusion criteria were as follows: (1) conference abstracts or study protocols; (2) duplicate publications; and (3) studies with incomplete data or no relevant outcome.

### Research Selection

Two reviewers (SL and XL) independently screened the literature. Initially, duplicate and irrelevant publications were excluded based on their title and abstract. Later, each of them independently read the full text of each potentially eligible article and finally identified all studies. In case of disagreement, discussions were conducted with a third investigator (JS Li) until consensus was reached.

### Data Extraction

Two reviewers (SL and XL) independently extracted the following data using predesigned forms according to the guideline for data extraction for systematic reviews and meta-analysis (Taylor et al., 2021), including the following information: first author, year of publication, country, study type, sample size, study period, follow-up years, age of participants, diagnosis of sepsis and dementia or cognitive impairment, sepsis type, dementia type, and confounder.

### Risk-of-Bias Assessment

The Newcastle-Ottawa Quality Assessment Scale (NOS) (Stang, 2010) was used to assess the quality of the included studies in three aspects, namely, selection, comparison, and results. The scores of cohort studies and case-control studies ranged from 0 to 9. Higher scores indicated a higher research quality; specifically, NOS scores of ≥ 7, 4–6, and 0–3 indicated high, medium, and low quality, respectively.

### Statistical Analysis

The Stata software (version 15.1) was used to conduct the data analysis. We extracted data on the adjusted OR and 95% CI from each study to assess the risk of dementia or cognitive impairment in sepsis survivals. We assessed heterogeneity using the chi-square test and *I*^2^ value, and *p* < 0.1 or *I*^2^> 50% was considered to indicate heterogeneity; in such instances, the random-effects model was adopted. Otherwise, the fixed-effects model was employed. We performed subgroup and sensitivity analyses to verify the robustness of the overall results and explore the sources of heterogeneity. The funnel plots and Egger’s regression test were used to detect the publication bias.

## Results

### Search Results

**FIGURE 1:**
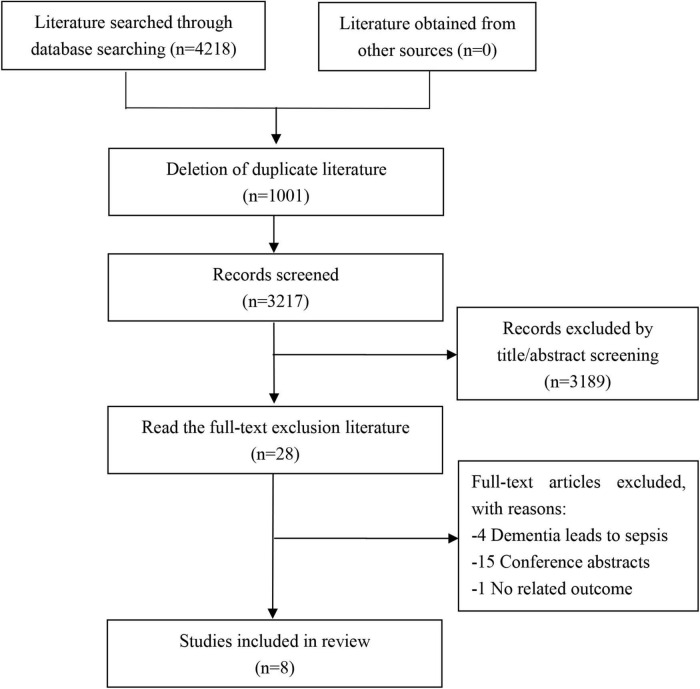
Literature screening flowchart.

We acquired a total of 4,218 articles from the initial search, of which 1,001 duplicate articles were excluded. Furthermore, 3,189 unrelated articles were excluded after the title and abstract screening and then 20 articles after full-text reading, including articles reporting dementia leading to sepsis (*n* = 4) (Shen et al., 2012; Kao et al., 2015; Liao et al., 2015; Bouza et al., 2019), conference abstracts (*n* = 15), and articles with no related outcome (*n* = 1) (Davydow et al., 2012). Finally, eight studies were included in this review. The search selection process is shown in Figure 1.

### Characteristics of Studies

We included eight studies (Iwashyna et al., 2010; Guerra et al., 2012; Shah et al., 2013; Mawanda et al., 2016; Chou et al., 2017; Sakusic et al., 2018; Ahlström et al., 2020; Fritze et al., 2020) involving a total of 891,562 individuals; of these studies, seven studies (Iwashyna et al., 2010; Guerra et al., 2012; Shah et al., 2013; Mawanda et al., 2016; Chou et al., 2017; Ahlström et al., 2020; Fritze et al., 2020) were cohort studies and one study (Sakusic et al., 2018) was a case-control study. These studies were published from 2010 to 2020. The sample size for each study ranged from 4,802 to 417,172 individuals, and the follow-up period ranged from 3 months to 11 years. Five studies (Iwashyna et al., 2010; Guerra et al., 2012; Shah et al., 2013; Mawanda et al., 2016; Sakusic et al., 2018) were conducted in the United States, one in China (Chou et al., 2017), one in Sweden (Ahlström et al., 2020), and one in Germany (Fritze et al., 2020). The mean age of the participants in the included studies ranged from 61 to 76.9 years. The International Classification of Diseases-9 (ICD-9) or International Classification of Diseases-10 (ICD-10) diagnostic codes were used as the diagnostic criteria for sepsis and dementia in six studies (Guerra et al., 2012; Shah et al., 2013; Mawanda et al., 2016; Chou et al., 2017; Ahlström et al., 2020; Fritze et al., 2020). The outcome measure was dementia (all types) in six studies (Guerra et al., 2012; Shah et al., 2013; Mawanda et al., 2016; Chou et al., 2017; Ahlström et al., 2020; Fritze et al., 2020) and cognitive impairment in two studies (Iwashyna et al., 2010; Sakusic et al., 2018). The main characteristics of the included studies are shown in Table 1.

**TABLE 1:** Characteristics of studies included in the review.

Authors	Country	Study type	Sample size	Subjects with outcome	Study period	Follow up years	Age (years)	Diagnosis of sepsis	Diagnosis of dementia/cognitive impairment	Sepsis type	Outcome	Confounders adjusted
**Dementia**												

Ahlström et al. (2020)	Swedish	Retrospective cohort	Total:210334 sepsis:16115, no sepsis:194219	Total:6312 Sepsis:472	2005–2015	11year (average 3.9)	61 average	ICD-10: A41.9, R65.1, R57.9	ICD-10 CCI: F00x-F03x, F051, and G30x-31x	Sepsis/Severe sepsis/Septic shock	All−cause dementia	Age, sex, CCI score, SAPS3 box2+3, Hospital LoS, ICU-LoS, invasive ventilator therapy, RRT
Chou et al. (2017)	China Taiwan	Retrospective cohort	Total: 61398 sepsis: 20466, no-sepsis: 40932	sepsis: 832, no-sepsis: 1945	2001–2011	/	Sepsis: 65.6 comparison: 65.4 average	ICD-CM(003.1, 036.2, and 038)	ICD-9-CM(290, 294.1 and 331.0)	Sepsis	All−cause dementia/AD/non- AD	Age, sex, stroke, DM, hyperlipidemia, hypertension, depression, ARD, smoking, and NSAID use
Guerra et al. (2012)	United States	Retrospective cohort	Total: 25368, sepsis:3145	Total:4519, sepsis:683	2005–2008	3	76.6 ± 6.8	ICD-9-CM	ICD-9-CM: (290.x, 294.x, 331.x, 797.x	Severe sepsis	All−cause dementia	Age, race, gender, cerebrovascular disease, Parkinson’s disease, alcohol abuse, hypertension, hypoglycemia and chronic renal failure
Mawanda et al. (2016)	United States	Retrospective cohort study	Total:417172	25639	2003–2012	9.03 ± 1.1	67.7 average	ICD-9	ICD-9: 290.0x, 290.10, 290.11, 290.12, 290.13, 290.20, 290.21, 290.3x, and 331.2; 290.40, 290.41, 290.42, and 290.43; 294.10, 294.11, and 294.8x; 331.0; 331.11 and 331.19 , and 331.82	Septicemia	All−cause dementia	Demographic characteristics (age, gender, race/ethnicity, and annual income), medical comorbidity and psychiatric covariates (traumatic brain injury, hypertension, ischemic heart disease, cerebrovascular disease, atherosclerosis, diabetes mellitus, chronic obstructive pulmonary disease, chronic kidney disease, chronic liver disease, peptic ulcer disease/gastritis, bipolar disorder, PTSD, schizophrenia, and alcohol abuse)
Shah et al. (2013)	United States	Prospective cohort study	total:5888, dementia assessed in 3602 participants	707	1997-to unknown follow up	over 10 years	72.8 ± 5.6	ICD-9	Neuropsychiatric testing, magnetic resonance imaging evaluations and annually with the (3MS) examination.	Severe sepsis	Dementia	Demographics, health behaviors, other chronic health conditions, trajectories of physical and cognitive decline before pneumonia hospitalization
Fritze et al. (2020)	Germany	Retrospective cohort	Total: 161567	/	2004–2015	/	Over 65	ICD- 10(A41, F05, F06)	ICD-10(G30, G31.0, G31.82, G23.1, F00, F01, F02, F03, F05.1)	Sepsis	All- cause dementia	Delirium, surgery, age, sex, and comorbidities

**Cognitive impairment**												

Iwashyna et al. (2010)	United States	Prospective cohort	Total: 5033 (516surviving with sepsis and 4517 surviving without sepsis)	623	1998–2006	Until 2006 or death, whichever occurred first	76.9 average	/	Combined score of ADLs and IADL	Severe sepsis	Moderate to severe cognitive impairment	/
Sakusic et al. (2018)	United States	Nested case-control study	Cases: 793/2401. Controls: 736/2401.	Cases: 793 . Control: 736.	2004–2015	3–24 months after ICU discharge	65.9 average	/	Manually reviewing electronic health records using algorithms for cognitive impairment and dementia	Sepsis	Persistent cognitive impairment	Charlson Comorbidity Index and N. of ICU stays

### Risk-of-Bias Assessment

The NOS scale was used to assess the quality of the included studies, and the results are shown in Table 2. Seven studies (Guerra et al., 2012; Shah et al., 2013; Mawanda et al., 2016; Chou et al., 2017; Sakusic et al., 2018; Ahlström et al., 2020; Fritze et al., 2020) had a score of ≥ 7 and were classified as having a high quality; one study (Iwashyna et al., 2010) had a score of 6 and was classified as having a moderate quality. The mean score of the seven studies was 7.375, indicating an overall high quality.

**TABLE 2:** Newcastle-Ottawa Quality Assessment Scale for cohort studies and case-control studies included in this review.

COHORT STUDIES				
References	Selection	Comparability	Outcome	Overall quality score
Ahlström et al. (2020)	★★★★	★★	★★★	9
Chou et al. (2017)	★★★★	★★	★	7
Guerra et al. (2012)	★★★	★	★★★	7
Iwashyna et al. (2010)	★★★	★	★★	6
Mawanda et al. (2016)	★★★★	★★	★★	8
Shah et al. (2013)	★★★	★★	★★	7
Fritze et al. (2020)	★★★★	★★	★★	8

**CASE-CONTROL STUDIES**				

**First author**	**Selection**	**Comparability**	**Exposure**	**Overall quality score**

Sakusic et al. (2018)	★★★	★★	★★	7

***Cohort Studies: Selection** ① Representativeness of the exposed cohort★ ② Selection of the non exposed cohort★ ① Ascertainment of exposure★ ④ Demonstration that outcome of interest was not present at start of study★.*

***Comparability** ① Comparability of cohorts on the basis of the design or analysis★★. **Outcome** ① Assessment of outcome★ ② Was follow-up long enough for outcomes to occur★ ③ Adequacy of follow up of cohorts★*

***Case Control Studies: Selection** ① Is the case definition adequate★★ ② Representativeness of the cases★ ③ Selection of Controls★ ④ Definition of Controls★. **Comparability** ① Comparability of cohorts on the basis of the design or analysis★★. **Outcome** ① Ascertainment of exposure★ ② Same method of ascertainment for cases and controls★ ③ Non-Response rate★.*

### Sepsis Survivals and Risk of All-Cause Dementia

**FIGURE 2:**
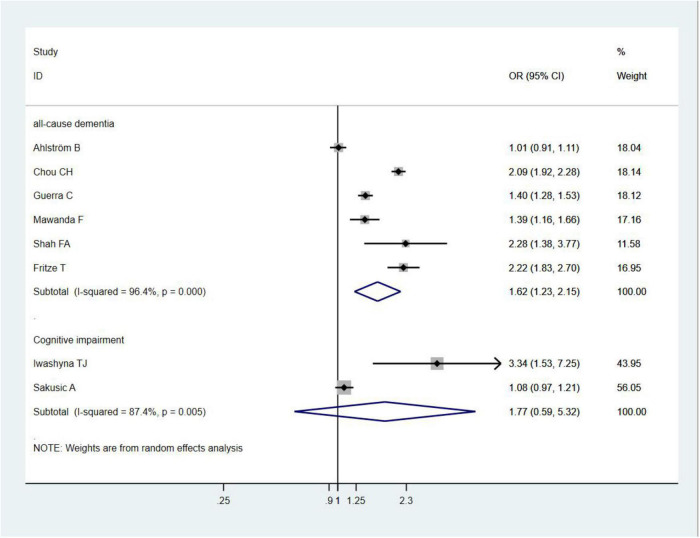
Forest plot showing the effect of sepsis on all-cause dementia or cognitive impairment.

**FIGURE 3:**
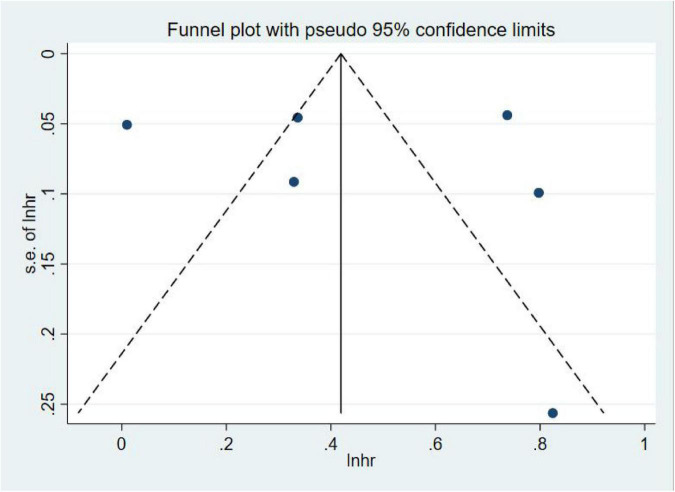
Funnel figure showing the effect of sepsis on all-cause dementia.

A total of six studies (Guerra et al., 2012; Shah et al., 2013; Mawanda et al., 2016; Chou et al., 2017; Ahlström et al., 2020; Fritze et al., 2020) assessed dementia as an outcome. Of them, five studies (Guerra et al., 2012; Shah et al., 2013; Mawanda et al., 2016; Chou et al., 2017; Fritze et al., 2020) showed that sepsis was associated with an increased risk of all-cause dementia, with effect estimates (OR) ranging from 1.39 (95% CI = 1.16–1.66) to 2.28 (95% CI = 1.38–3.77); one study (Ahlström et al., 2020) showed that sepsis was not associated with an increased risk of dementia (OR = 1.01, 95% CI = 0.91–1.11). In general, sepsis survivals were associated with an increased risk of all-cause dementia (OR = 1.62, 95% CI = 1.23–2.15, *I*^2^ = 96.4%, *p* = 0.001; Figure 2). Owing to the significant heterogeneity (*I*^2^ = 96.4%, *p* = 0.000), we performed a sensitivity analysis by omitting each study to explore the source of heterogeneity and found that the overall results were relatively robust (Supplementary Appendix 2). A visual inspection of the funnel plots yielded no evidence of a significant publication bias (Figure 3). The Egger’s regression tests (*p* = 0.786) indicated a low possibility of publication bias in this meta-analysis.

### Gender of Sepsis Survivals and Risk of Dementia

**FIGURE 4:**
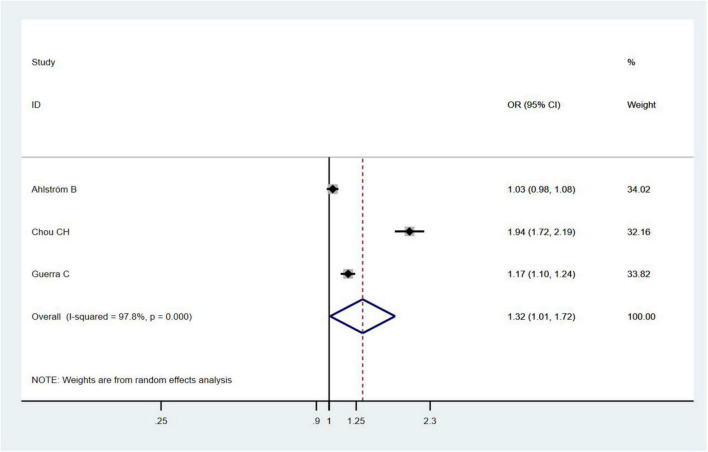
Funnel figure showing the effect of gender of sepsis survivals on dementia.

Three studies (Guerra et al., 2012; Chou et al., 2017; Ahlström et al., 2020) assessed the relationship between the gender of sepsis survivals and risk of dementia and found that female sepsis survivals had an increased risk of dementia (OR = 1.32, 95% CI = 1.01–1.92, *I*^2^ = 97.8%, *p* = 0.043; Figure 4).

### Sepsis Survivals and Risk of Cognitive Impairment

Two studies (Iwashyna et al., 2010; Sakusic et al., 2018) evaluated the risk of cognitive impairment in sepsis survivals and found that sepsis was not associated with an increased risk of cognitive impairment (OR = 1.77, 95% CI = 0.59–5.32, *I*^2^ = 87.4%, *p* = 0.306; Figure 2).

### Types of Sepsis and Risk of Dementia or Cognitive Impairment

**FIGURE 5:**
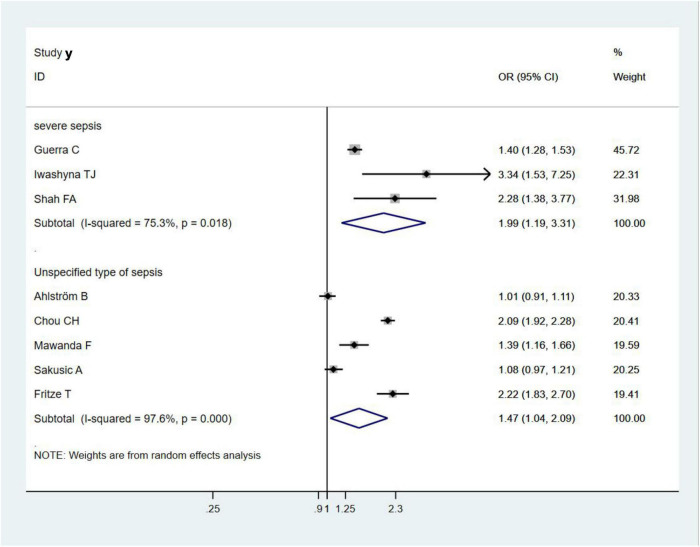
Types of sepsis and risk of dementia or cognitive impairment.

Three studies (Iwashyna et al., 2010; Guerra et al., 2012; Shah et al., 2013) evaluated the association between severe sepsis and the risk of dementia or cognitive impairment and found that severe sepsis survivals had an increased risk of dementia or cognitive impairment (OR = 1.99, 95% CI = 1.19–3.31, *I*^2^ = 75.3%, *p* = 0.008; Figure 5). Similarly, the other five included studies showed that other unspecified types of sepsis were associated with an increased risk of dementia or cognitive impairment (OR = 1.47, 95% CI = 1.04–2.09, *I*^2^ = 97.6%, *p* = 0.029; Figure 5); however, the risk was significantly lower than that in severe sepsis.

## Discussion

In this systematic review and meta-analysis of observational studies, we compiled current evidence on the association between sepsis survivals and the risks of dementia or cognitive impairment in 891,562 individuals from eight population-based studies (Iwashyna et al., 2010; Guerra et al., 2012; Shah et al., 2013; Mawanda et al., 2016; Chou et al., 2017; Sakusic et al., 2018; Ahlström et al., 2020; Fritze et al., 2020). We found that sepsis survivals had an increased risk of all-cause dementia. In contrast, a lack of association between sepsis and the risk of cognitive impairment was observed in two studies (Iwashyna et al., 2010; Sakusic et al., 2018). Subgroup analyses showed that severe sepsis was associated with a higher risk of dementia or cognitive impairment than other unspecified types of sepsis.

This study found that sepsis survival could increase the risk of dementia, which is consistent with the results of previous studies. Muzambi et al. (2020) showed that common bacterial infections, such as pneumonia, urinary tract infection, and cellulitis, played a role in increasing the risk of dementia. Furthermore, their subgroup analysis indicated that sepsis survival was associated with a 1.39–2.60-fold higher risk of dementia than non-sepsis patients, which was higher than that observed in our study. This finding may be partly attributed to the increased risk of sepsis in patients with dementia who were included in their review, which may have contributed to excessive results. Meanwhile, Bouza et al. (2019) found that 16,829 (11.3%) of 148,293 patients with confirmed sepsis were diagnosed with dementia; of them, women and elderly patients were more often diagnosed, and patients were more frequently admitted to hospital because of sepsis. However, Bouza et al. (2019) failed to calculate the increased risk of dementia in relation to sepsis. In addition, a nested case-control study (Dunn et al., 2005) suggested that infection was associated with an increased risk of dementia but only assessed the overall effect of infection on dementia rather than the individual effect of each infection on dementia; we were unable to extract data from this study and, therefore, did not include such in this review. In contrast, more recent studies were added in the current analysis, thus providing robust evidence on the association between sepsis survival and the risk of dementia.

Consistent with previous findings, it is noteworthy that no significant association was found between sepsis survival and the risk of cognitive impairment in our meta-analysis (Muzambi et al., 2020). However, the result was based on only two studies, which may reduce statistical efficiency. Nevertheless, the systematic review by Calsavara et al. (2018) that included 16 studies reported that post-sepsis cognitive impairment was observed in 12.5–21% of sepsis survivals and that depressive symptoms, central nervous system infection, and length of hospitalization owing to infection were major risk factors for the development of dementia in sepsis survivals. Regrettably, Calsavara et al. (2018) did not perform a pooled analysis of sepsis survivals at an increased risk for cognitive impairment. This result is contrary to our review and may be related to the inconsistency in the sample size and inclusion criteria.

Subgroup analyses showed that severe sepsis was associated with a higher risk of developing dementia than other unspecified types of sepsis, which is largely consistent with the results suggested by Muzambi et al. (2020). Their study found that as the severity of sepsis increased from mild to severe, the risk of dementia increased from 1.20- to 5.04-fold. Similarly, another study (Chou et al., 2018) also showed an increase in the risk of dementia as the severity of sepsis increased. However, owing to the small number of studies included in this review, we were unable to further investigate the effects of severity, frequency, and timing of infection on the risk of dementia.

Only two of all included studies (Sakusic et al., 2018; Ahlström et al., 2020) showed that sepsis survival had no increased risk of dementia or cognitive impairment. We speculated that this may be related to the following reasons: the sample size included in the study varies greatly, which may have an impact on the results. Furthermore, the different confounders adjusted by each study could be another reason. In addition, Ahlström et al. (2020) excluded dementia diagnoses registered in the first year after intensive care unit (ICU) admission, which may have missed some data and, thus, had an impact on the overall results. Meanwhile, the diagnoses in the study by Sakusic et al. (2018) relied on follow-up information from 3 months to 2 years of ICU stay, which may have underestimated the proportion of patients with cognitive impairment.

The pathophysiological mechanism of the association between sepsis and dementia or cognitive impairment remains largely unclear (Itzhaki et al., 2016; Fulop et al., 2018); nevertheless, several mechanisms are speculated to be involved. First, pro-inflammatory mediators can cross the blood-brain barrier and activate cytotoxic microglia when systemic inflammation occurs, which could lead to deterioration of cognitive function and increase the risk of dementia (Weberpals et al., 2009; Sochocka et al., 2017; Rocha et al., 2021). Second, as sepsis is induced in animal models, it triggers systemic inflammation, leading to the accumulation of amyloid-β and cognitive dysfunction (Sundelöf et al., 2009). In addition, studies have found that the microglia become over-responsive to stress or stimuli as age increases, generate neurotoxic and pro-inflammatory molecules, and accelerate the development of age-related diseases, such as dementia or cognitive impairment (Olivieri et al., 2018). Sonneville et al. (2013) found that sepsis was characterized by an acute brain dysfunction whose mechanisms included excessive microglial activation, impaired cerebral perfusion, blood-brain barrier dysfunction, and altered neurotransmission, so modulation of microglial activation, prevention of blood-brain barrier alterations, and use of antioxidants represented relevant therapeutic targets that may impact significantly on neurologic outcomes in the future. Lopez-Rodriguez et al. (2021) revealed that acute systemic inflammation may trigger a neuroinflammatory phenotype switch, with interleukin-1β (IL-1β) driving amplified responses in primed microglia, astrocytes, and neuronal network dysfunction which exacerbated neuroinflammation in Alzheimer’s disease. Notably, most of the current studies on the relationship between sepsis and dementia or cognitive impairment utilized rodent models, and there are few studies on human cases. Thus, the potential pathological mechanism needs to be further explored.

The heterogeneity of the included studies was large, which may be attributed to the following reasons. The experimental data of the studies included in the analysis were small, and only two studies analyzed the relationship between sepsis and the risk of cognitive impairment, which might have affected the accuracy of the results. Therefore, studies with larger sample sizes and more cohort studies with long-term follow-up are needed to clarify the association. Furthermore, the diagnostic criteria of sepsis and dementia were inconsistent, such as ICD-9 or ICD-9-CM, or ICD-10, and the disease diagnosis mainly depended on electronic health records, which may lead to the deviation of results. In addition, the different confounders adjusted for each study may be another source of heterogeneity.

To the best of our knowledge, our review is the first meta-analysis to include only studies of sepsis survivals and the risk for dementia or cognitive impairment, which may contribute to the accurate assessment of whether sepsis survivals are associated with an increased risk of dementia or cognitive impairment. Admittedly, there were several limitations in our study. First, the number of included studies was limited; thus, we could not perform subgroup analyses on the type of dementia to demonstrate the risk of different types of dementia in sepsis survivals. Second, the included studies showed significant heterogeneity in terms of study design, population size, and definitions of sepsis, dementia, and cognitive impairment. Therefore, the potential effect of heterogeneity on the results should be taken into account when interpreting our results. Third, the generalizability of the findings of the included studies should be concerned. As the studies were conducted in the United States, China, Germany, and Sweden, regional bias is a valid possibility.

## Conclusion

Sepsis survivals are associated with an increased risk of all-cause dementia but not with cognitive impairment. Appropriate management and prevention are essential to preserve the cognitive function of sepsis survivals and reduce the risk of dementia.

## Data Availability Statement

The original contributions presented in the study are included in the article/Supplementary Material, further inquiries can be directed to the corresponding author/s.

## Author Contributions

JL and XL contributed to design and conception of the article. SL and XL contributed to collection and assembly of materials, and drafted the manuscript. SL, XL, HZ, ZF, and LC contributed to data interpretation and analysis. XL, HZ, ZF, LC, YX, and JL revised the manuscript. All authors reviewed and approved the final version of the manuscript.

## Conflict of Interest

The authors declare that the research was conducted in the absence of any commercial or financial relationships that could be construed as a potential conflict of interest.

## Publisher’s Note

All claims expressed in this article are solely those of the authors and do not necessarily represent those of their affiliated organizations, or those of the publisher, the editors and the reviewers. Any product that may be evaluated in this article, or claim that may be made by its manufacturer, is not guaranteed or endorsed by the publisher.

## Abbreviations

AD: Alzheimer’s disease
ICD: International Classification of Diseases
3MS: Modified Mini-Mental State Examination
ICU: Intensive Care Unit
PTSD: post-traumatic stress disorder
DM: diabetes mellitus
ARD: alcoholism-related disease
NSAID: non-steroidal anti-inflammatory drug
LoS: Length of stay
SAPS3: Simplified Acute Physiology Score 3
CCI: Charlson Comorbidity Index
RRT: renal replacement therapy.
