# Supplementary material for: Risk of Dementia or Cognitive Impairment in Sepsis Survivals: A Systematic Review and Meta-Analysis
Source: Front Aging Neurosci. 2022 Mar 9;14:839472. doi: 10.3389/fnagi.2022.839472 (PMC8959917; doi:10.3389/fnagi.2022.839472)
Supplement: Supplementary file 1 [file Table_1.doc]

**Supplementary table 1**

**Details of the Literature Search Strategy**

(1) PubMed

| **Search** | **Query** | **Items found** |
| --- | --- | --- |
| #1 | (("Sepsis"[Mesh]) OR "Shock, Septic"[Mesh]) | 131857 |
| #2 | (((((((Sepsis*[Title/Abstract]) OR (Septicemia*[Title/Abstract])) OR (Septic shock*[Title/Abstract])) OR (Severe sepsis*[Title/Abstract])) OR (Systemic Inflammatory Response Syndrome[Title/Abstract])) OR (SIRS[Title/Abstract])) OR (septic[Title/Abstract])) OR (septicaemic shock[Title/Abstract]) | 163176 |
| #3 | #1OR#2 | 232797 |
| #4 | ("Dementia"[Mesh]) OR "Alzheimer Disease"[Mesh] | 182893 |
| #5 | (((((Dementia[Title/Abstract]) OR (Alzheimer's disease[Title/Abstract])) OR (Cognitive decline[Title/Abstract])) OR (cognitive impairment[Title/Abstract])) OR (cognitive disorder[Title/Abstract])) OR (cognitive dysfunction[Title/Abstract]) | 278910 |
| #6 | #4 OR #5 | 326587 |
| #7 | #3 AND #6 | 839 |

(2) Embase

| **Search** | **Query** | **Items found** |
| --- | --- | --- |
| #1 | 'sepsis'/exp | 302509 |
| #2 | 'septic shock'/exp | 63799 |
| #3 | sepsis*:ti OR septicemia*:ti OR 'septic shock*':ti OR 'severe sepsis*':ti OR 'systemic inflammatory response syndrome':ti OR sirs:ti OR septic:ti OR 'septicaemic shock':ti | 81353 |
| #4 | #1 OR #2 OR #3 | 315769 |
| #5 | 'dementia'/exp | 395081 |
| #6 | 'alzheimer disease'/exp | 218076 |
| #7 | dementia:ti OR 'alzheimers disease':ti OR 'cognitive decline':ti OR 'cognitive impairment':ti OR 'cognitive disorder':ti OR 'cognitive dysfunction':ti | 110431 |
| #8 | #5 OR #6 OR #7 | 421523 |
| #10 | #4 AND #8 | 1850 |

(3) Cochrane Library

| **Search** | **Query** | **Items found** |
| --- | --- | --- |
| #1 | MeSH descriptor: [Sepsis] explode all trees | 4756 |
| #2 | MeSH descriptor: [Shock, Septic] explode all trees | 1034 |
| #3 | (Sepsis*):ti,ab,kw OR (Septicemia*):ti,ab,kw OR (Septic shock*):ti,ab,kw OR (Severe sepsis*):ti,ab,kw OR (Systemic Inflammatory Response Syndrome):ti,ab,kw OR (SIRS):ti,ab,kw OR (septic):ti,ab,kw OR (septicaemic shock):ti,ab,kw | 16816 |
| #4 | #1 OR #2 OR #3 | 18371 |
| #5 | MeSH descriptor: [Dementia] explode all trees | 6353 |
| #6 | MeSH descriptor: [Alzheimer Disease] explode all trees | 3607 |
| #7 | (Dementia):ti,ab,kw OR (Alzheimer's disease):ti,ab,kw OR (Cognitive decline):ti,ab,kw OR OR (cognitive impairment):ti,ab,kw OR (cognitive disorder):ti,ab,kw OR (cognitive dysfunction):ti,ab,kw | 60959 |
| #7 | #5 OR #6 OR #7 | 61181 |
| #8 | #4 AND #7 | 183 |

(4) [Web of Science](https://apps.webofknowledge.com/home.do?SID=6BQQjiiMCVa9MgFvRpC) core collection

| **Search** | **Query** | **Items found** |
| --- | --- | --- |
| #1 | Topic: (Sepsis*) OR Topic: (Septicemia*) OR Topic: (Septic shock*) OR Topic: (Severe sepsis*) OR Topic: (Systemic Inflammatory Response Syndrome) OR Topic: (SIRS) OR Topic: (septic) OR Topic: (septicaemic shock) | 215974 |
| #2 | Topic: (Dementia) OR Topic: (Alzheimer's disease) OR Topic: (Cognitive decline) OR Topic: (cognitive impairment) OR Topic: (cognitive disorder) OR Topic: (cognitive dysfunction) | 494844 |
| #3 | #1 AND #2 | 1346 |
