# Supplementary material for: Risk of Dementia or Cognitive Impairment in Sepsis Survivals: A Systematic Review and Meta-Analysis
Source: Front Aging Neurosci. 2022 Mar 9;14:839472. doi: 10.3389/fnagi.2022.839472 (PMC8959917; doi:10.3389/fnagi.2022.839472)
Supplement: Supplementary file 2 [file Table_2.doc]

**Supplementary table 2**

**Sensitivity Analysis**


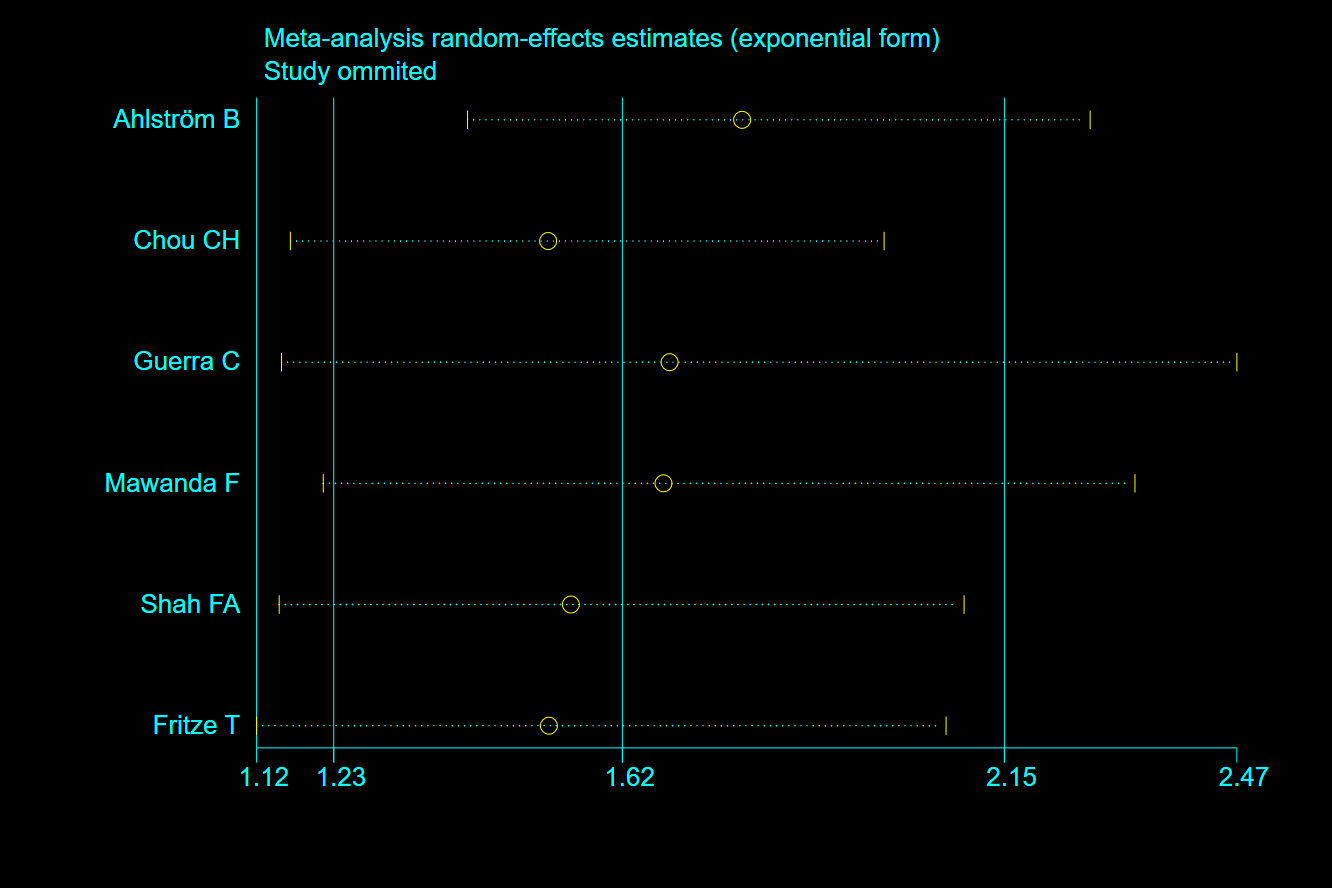


Figure 1 –Sensitivity analysis showing the effect of sepsis on all-cause dementia

Table 1 Sensitivity analysis showing the effect of sepsis on all-cause dementia

| Deletion | Result |
| --- | --- |
| Ahlström B | OR=1.79, 95% CI [1.41, 2.26] |
| Chou CH | OR=1.52, 95% CI [1.17, 1.98] |
| Guerra C | OR=1.69, 95% CI [1.15, 2.47] |
| Mawanda F | OR=1.68, 95% CI [1.21, 2.33] |
| Shah FA | OR=1.55, 95% CI [1.15, 2.09] |
| Fritze T | OR=1.52, 95% CI [1.12, 2.07] |
