# Supplementary material for: Risk of Dementia or Cognitive Impairment in Sepsis Survivals: A Systematic Review and Meta-Analysis
Source: Front Aging Neurosci. 2022 Mar 9;14:839472. doi: 10.3389/fnagi.2022.839472 (PMC8959917; doi:10.3389/fnagi.2022.839472)
Supplement: Supplementary file 3 [file Table_3.docx]

**Supplementary table 3**

**Characteristics of Excluded Studies**

| **Author, Year** | **Tittle** | **Reasons** |
| --- | --- | --- |
| Beesley, S,  2018 | Cognitive impairment after sepsis (CASS) study: Predictors of cognitive impairment | Conference abstracts |
| Castilla-Puentes, R. C, 2014 | Co-morbid medical conditions in vascular dementia: A case-control study |  |
| Chou, C. H,  2018 | Association between septicemia and vascular dementia: A nation-wide population-based study |  |
| Christelle, M,  2018 | Early identification of sepsis-associated encephalopathy with EEG is not associated with short-term cognitive dysfunction |  |
| Ehlenbach, W. J,  2015 | Association between severe sepsis and microvascular brain injury in a prospective cohort study |  |
| Falade, O,  2014 | Emergence of new health conditions in severe sepsis and infection survivors |  |
| Koami, H,  2018 | Cognitive impairment is one of the predictors of long-term mortality of septic patients |  |
| Kurtz, P,  2016 | Brain dysfunction in severe sepsis: an observational study |  |
| Paratz, J,  2016 | A follow up clinic for sepsis survivors-preliminary results and feasibility |  |
| Rakic, S,  2016 | The effects of acute systemic infection on Alzheimer's disease |  |
| Sakusic, A,  2018 | Risk factors for persistent cognitive impairment after critical illness: Nested case control study |  |
| Santoro, M. J,  2011 | Cognitive impairment at discharge in intensive care unit (ICU) Survivors: Data from the awakening and breathing controlled (ABC) trial |  |
| Shah, F. A,  2012 | Trajectories of physical disability and cognitive decline and the risk of severe sepsis hospitalizations in older adults: The cardiovascular health study |  |
| Wang, H. E,  2020 | Sepsis accelerates long-term trajectories of cognitive decline and impairment |  |
| Widmann, C,  2012 | Sepsis and cognition |  |
| Kao LT,  2015 | Association between sepsis and dementia. | Dementia leads to sepsis |
| Liao KM,  2015 | Dementia Increases Severe Sepsis and Mortality in Hospitalized Patients With Chronic Obstructive Pulmonary Disease. |  |
| Shen HN,  2012 | Dementia increases the risks of acute organ dysfunction, severe sepsis and mortality in hospitalized older patients: a national population-based study. |  |
| Bouza C,  2019 | The impact of dementia on hospital outcomes for elderly patients with sepsis: A population-based study. |  |
| Davydow DS,  2012 | Presepsis depressive symptoms are associated with incident cognitive impairment in survivors of severe sepsis: a prospective cohort study of older Americans. | No related outcome |
